# Lifetime relapse and its associated factors among people with schizophrenia spectrum disorders who are on follow up at Comprehensive Specialized Hospitals in Amhara region, Ethiopia: a cross-sectional study

**DOI:** 10.1186/s13033-021-00464-0

**Published:** 2021-05-06

**Authors:** Solomon Moges, Tilahun Belete, Tesfa Mekonen, Melak Menberu

**Affiliations:** 1 College of Health Sciences, Department of Psychiatry, Woldia University, Woldia, Ethiopia; 2 College of Medicine and Health Sciences, Department of Psychiatry, Bahir Dar University, Bahir Dar, Ethiopia

**Keywords:** Relapse, Schizophrenia spectrum disorders, Amhara, Ethiopia

## Abstract

**Background:**

Relapse in psychiatric disorders is highly distressing that posed a huge burden to the patients, family, and society. It interrupts the process of recovery and may increase the risk of resistance to treatment. Relapse detection and taking preventive measures against its possible factors are crucial for a better prognosis.

**Objective:**

To assess lifetime relapse and its associated factors among people with schizophrenia spectrum disorders who are on follow-up at Comprehensive Specialized Hospitals in Amhara region, Ethiopia.

**Method:**

An institution-based cross-sectional study was conducted from July 13-August 13, at Comprehensive Specialized Hospitals in Amhara region, Ethiopia, 2020. Data were collected from 415 randomly selected participants using an interviewer administered questionnaire. Relapse was determined using participants’ medical records and a semi-structured questionnaire. Data were analyzed using Statistical Package for Social Sciences (SPSS) version 25. Logistic regression analysis was done to identify the explanatory variables of relapse. Variables with P-value < 0.05 were considered significantly associated with relapse.

**Result:**

The magnitude of lifetime relapse was 57.4% (95% CI = 53–62%). Relapse was significantly associated with comorbidity of another mental illness (AOR = 1.84, 95% CI = 1.06, 3.18), non-adherence to medication (AOR = 2.23, 95% CI = 1.22, 4.07), shorter duration on treatment (AOR = 1.71, 95% CI = 1.05, 2.81), and experiencing stressful life events (AOR = 2.42, CI = 1.2, 4.66).

**Conclusion:**

In the current study, more than half of the participants had lifetime relapses. Comorbid mental illnesses, non-adherence, duration of treatment ≤ 5 years, and experiencing stressful life events were factors associated with relapse. This requires each stakeholder to give concern and work collaboratively on the respective factors that lead to relapse.

## Introduction

Mental disorders comprised the highest global disease burden where relapse is one of the most important barriers to recovery and rehabilitation. Relapse in schizophrenia spectrum disorders is an issue of concern to both the patients and caregivers causing high distress and poor quality of life [1–4]. Relapse in schizophrenia spectrum disorders refers to a return of symptoms after a period of improvement which is characterized by acute psychotic exacerbation [5, 6]. It is associated with frequent hospitalizations and contributes to considerable costs in mental healthcare [7, 8].

In the course of schizophrenia spectrum disorders, relapse is a common phenomenon even while the patient is on treatment. For instance, the relapse rate for people with schizophrenia ranges between 50 to 92% globally and is estimated to be 3.5% per month in those who are treated with depot antipsychotic medication [9, 10].

The first two to five years are thought to be key determinants of long-term functional and clinical prognosis associated with schizophrenia. Rates of relapse in psychotic disorder were 31% after one year and 43% after two years of treatment [11]. In another study, the relapse rate within the first years after schizophrenia onset has been estimated to be about 34– 37% [12], while the lifetime risk of relapse was up to 70%, irrespective of the pharmacological treatment [13]. In a systematic review and meta-analysis of longitudinal studies in patients with first-episode psychosis, the pooled prevalence of relapse of positive symptoms was 28% within 1 year of follow-up, 43% within 1.5 to 2 years, and 54% within 3 years of the follow-up period [14]. As a result, with an increase in the number of relapses, the risk of chronicity of the disorder with severe functional impairment seems to be higher [15].

Frequent relapses have been associated with progressive functional deterioration, hospitalizations, cognitive impairment, and poor clinical prognosis, which further leads to an enormous burden for the patients and their families [16–18]. Additionally, relapse increases the risks of self-harm and social impairment [4].

Previous studies reported some of the modifiable risk factors associated with relapse, including poor treatment compliance, substance use, poor social support, delay in seeking care, stigma against mental illness, and unavailability of psychotropic drugs [19, 20]. Identifying the factors associated with relapse will help to predict and prevent relapse in patient care. However, evidence on the magnitude and associated factors of relapse is scarce in Ethiopia [10, 21, 22]. Therefore, the purpose of this study was to determine the magnitude of lifetime relapse and its associated factors among people with schizophrenia spectrum disorders.

## Methods

### Study design and setting

An institution-based cross-sectional study was conducted from July 13-August 13, 2020 at Comprehensive Specialized Hospitals located in Amhara region, Ethiopia, specifically in Felege Hiwot Comprehensive Specialized Hospital (FHCSH), University of Gondor Comprehensive Specialized Hospital (UGCSH), and Dessie Comprehensive Specialized Hospitals (DCSH).

FHCSH is located in Bahir Dar city 521 kms away from Addis Ababa, the capital city of Ethiopia. FHCSH psychiatry unit has seven inpatient beds and six outpatient departments. The total patient population served by the FHCSH Psychiatry unit is 19,200 per year, and currently, about 445 people with schizophrenia spectrum disorders are estimated to be seen monthly.

UGCSH is located in Gondar Town 730 kms away from Addis Ababa. The total number of patients served by UGCSH Psychiatry unit is 12,000 per year. UGCSH Psychiatry unit has two beds in the emergency, nineteen beds for inpatient services, and four outpatient departments. Currently, 378 people with schizophrenia spectrum disorders is estimated to be seen monthly.

DCSH is located 401 kms from Addis Ababa. The DCSH Psychiatry unit serves 15,100 patients per year. It has three outpatient departments and one inpatient service which has six beds and currently, about 433 patients with schizophrenia spectrum disorders are estimated to be seen.

### Source populations and study populations

All people who have a diagnosis of schizophrenia spectrum disorders according to DSM-5 and who are on follow up at Comprehensive Specialized Hospitals in the Amhara region, Ethiopia were the source population. The study population were all people with schizophrenia spectrum disorders aged 18 years and above who were receiving follow-up services and present during the data collection period at these hospitals. We included participants who were receiving follow-up service for at least six months duration because symptomatic remission is expected to be achieved within six months of starting medications/follow-up [23].

### Sample size and sampling procedure

**Fig. 1:**
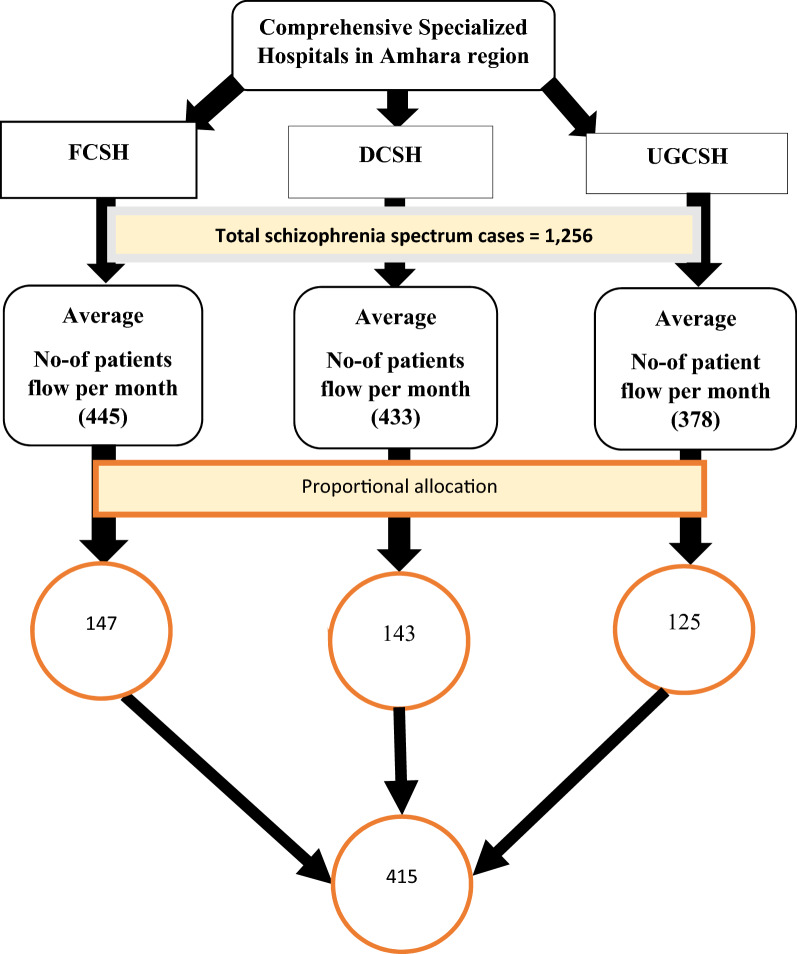
Sampling procedure of selecting study samples from all study areas, Amhara region, Ethiopia, 2020

The sample size was calculated using a single population proportion formula considering 43.3% relapse [19], 95% confidence interval, 5% margin of error, and 10% non-response rate. The final sample size was 415. Systematic random sampling technique was used to select participants using the list of patient cards as a sampling frame at the outpatient department (OPD) level. The final sample size was allocated proportionally for the three hospitals based on their monthly case flow. Sampling fraction (K) was determined by dividing the total study subjects who had a follow-up during the four-week data collection period (which was determined by taking the average monthly patient flow in the past 3 months) by each respective designated sample size **((445/147, 433/143, 378/125)≈**3): then, the first subject was selected randomly using lottery method. Until the completion of the final sample size, participants were selected every 3^rd^ interval (See Fig. 1 below).

### Study variables

The dependent variable was the lifetime relapse of schizophrenia spectrum disorders (yes/no). Independent variables included were socio-demographic related factors ( age, sex, marital status, religion, educational status, economic status, employment status, living condition); Clinical and psychosocial factors (class of antipsychotics medication, antipsychotic medication combination, medication non-adherence, medication side effects, duration of treatment, co-morbid psychiatric illnesses, comorbidity of physical/surgical illnesses, stressful life events, delay in treatment and social support); Substance-related factors (use of alcohol, khat chewing, cigarette smoking, cannabis use, cocaine, and heroin).

### Data collection and measurement tools

Face-to-face interviews using semi-structured questionnaires which consist of socio-demographic factors, clinical and psychosocial factors, substance-related factors, and document review were done to collect the data. The data were collected by six Bachelor of Science holders in Psychiatry. The data were collected from the patient and the caregivers when the patient did have an impaired insight. Data collection was supervised by 1 Master of Science holder in Psychiatry profession in each study site; those were recruited during data collection time.

Relapse was determined based on the participant’s medical record review and interview with the patient and their caregivers using a semi-structured questionnaire. In addition to the participant’s medical record review, relapse was identified among people with schizophrenia spectrum disorders in any of the following cases within six to twelve months after remission (re-emergence or aggravation of psychotic symptoms, consultation with a psychiatrist/ more intensive case management, medication change for deterioration of illness, and admission to a psychiatric unit [2, 4]). Medication Adherence Rating Scale (MARS) was used to measure medication adherence [24], which is a ten-item yes/no self-report instrument with a good internal consistency (α = 0.86). This tool was developed from the 30-item Drug Attitudes Inventory scale [25], and the 4-item Medication Adherence Questionnaire [26]. The total scores of MARS range from 0 (low likelihood of medication adherence) to 10 (high likelihood) [27]. The MARS was selected as a measure of medication adherence in the Psychological Prevention of Relapse in Psychosis trial [28]. MARS has been used in Ethiopia [29, 30].

Antipsychotic medication side effects were assessed using the 22-item, modified version of Glasgow Antipsychotic Side effect Scale (GASS). Side effects assessed by the GASS are sedation/cognition, cardiovascular side effects, extrapyramidal symptoms, anticholinergic side effects, gastrointestinal, genitourinary side effects, screening of diabetes mellitus, prolactin/endocrine side effects, and weight gain. The extent of side effects is rated from none (0) to every day (3 points) for questions 1–20 and no (0) and yes (3 points) for questions 21–22. Sensitivity ranged from 33–96% and the test–retest reliability is substantial (kappa = 0.72) [31].

Social support was measured by using the Oslo Social Support Scale (OSSS-3) [32]. The OSSS-3 total score ranges from 3–14. Scores from 3 to 8 are considered to indicate poor support, scores from 9 to 11 indicate intermediate support, and a score between 12 and 14 is considered to indicate strong social support. It has acceptable internal consistency (α = 0.640). This tool has been also used in Ethiopia settings [33–35].

The List of Threatening Experiences (LTE), with 12 categories of adverse life events, was used for assessing stressful life experiences in the past six months [36]. The LTE has good test–retest reliability (Kappa = 0.61–0.87) and predictive validity and low internal consistency (α = 0.44), which is also adapted and used in Ethiopia [37].

Alcohol abuse was assessed using the cut-annoyed-guilty-eye opening questionnaire (CAGE questionnaire), which has four items. Scoring of two or greater positive answers from the CAGE questionnaire was considered as having problematic alcohol use [38]. CAGE has an excellent test–retest reliability (0.80–0.95), a fair correlations (α = 0.48–0.70), and with an average sensitivity 0.71, specificity 0.90 [38]. Another substance abuse was measured using the Drug Abuse Screening Test (DAST-10), which is a 10-item asking about abuse, dependence, and low-risk substance use in the last 12 months. This measure has been included in previous research conducted in Ethiopia [19, 39–41]. DAST-10 has a very good internal consistency (α = 0.92), sensitivity, and specificity of 0.98 and 0.91 respectively [42]. Lifetime and current tobacco use were assessed using the first two items of the Alcohol, Smoking, and Substance Involvement Screening Test (ASSIST). ASSIST was developed by the World Health Organization with fair reliability (α = 0.73) [43].

### Data quality control

The questionnaire was prepared in English and translated into the Amharic language and then back-translated into English by two experts to ensure consistency and understandability of the tool. The instruments were pre-tested in 5% [21] of sample size at Tibebe Ghion Specialized Hospital. The final version of the questionnaire was established following the feedback from the pretest. The training was given to data collectors and supervisors by the principal investigator regarding the questioners’ detail, methods of data collection, quality control, and ethical consideration. The questionnaire was checked for its reliability and understandability. Supervision was carried out by supervisors at each site. After data collection, the filled questionnaires were checked for completeness and consistency.

### Data analyses

The data were edited, cleaned, coded, and entered into the computer using Epi-Data version 4.6 and analyzed using Statistical Package for Social Sciences (SPSS) version 25. The presence of an association between dependent and independent variables was assessed using logistic regression analysis. Variables with a p-value less than 0.2 at univariable logistic regression were entered into multivariable logistic regression. Statistical significance was considered at a p-value less than 0.05, and the strength of association was estimated in the odds ratio with a 95% confidence interval. Descriptive statistics including frequencies, percentages, and median was used to describe findings. Chi-square and odds ratio were done to determine the association of variables.

## Results

### Socio demographic characteristics of participants;

In the current study, a total of 404 (55.7% male) people with schizophrenia spectrum disorders were participated, yielding a response rate of 97.3%. The median age of the participant was 35 years with an interquartile range of 28–41 years. Among all, 179 (44.3%) of the participants were widowed, followed by a single 144 (35.6%). Regarding occupational status, 135 (33.4%) of the participants were farmers. The median monthly income of the participants was 2000 Ethiopian Birr (1 USD = 35.86 ETB) with an interquartile range of 0.00 to 3552.50 ETB. (See Table 1 below).

**Table 1:** Description of socio-demographic characteristics of participants with schizophrenia spectrum disorders in Comprehensive Specialized Hospitals, Amhara region, Ethiopia, 2020 (n = 404)

Variables		Frequency	Percentage (%)
Sex			
Male		225	55.7
Female		179	44.3
Age			
18–24		38	9.4
25–34		161	39.9
35–44		128	31.7
≥ 45		77	19
Marital status			
Single		144	35.6
Married		68	16.8
Divorced		13	3.3
Widowed		179	44.3
Religion			
Orthodox Christian		230	56.9
Muslim		163	40.3
Protestant		7	1.8
Others*		4	1
Residence			
Urban		254	62.9
Rural		150	37.1
Educational status			
Unable to read and write		76	18.8
Primary level		129	31.9
Secondary level		116	28.7
College and above		83	20.6
Occupational status			
Farmer		135	33.4
Merchant		58	14.4
Government and private worker		94	23.3
House wife		86	21.2
Others**		31	7.7
Living status			
Alone		57	14.1
Family		312	77.2
Relatives		16	4
Others***		19	4.7
Mean monthly income			
< 2044 ETB		203	50.2
≥ 2044 ETB		201	49.8

* = Jehovah’s Witness ** = Student, jobless, daily labor *** = Children, employer ETB = Ethiopian birr

### Clinical and psychosocial factors of participants

From the total participants, 346 (85.6%) were diagnosed with schizophrenia disorder. The median age of onset of the disorder was 29 years with an interquartile range of 23 to 34 years. The median treatment delay of the participants was 6 months with an interquartile range of 1.00 to 15.75 months. Nearly three fourth of the participants, 302 (74.8%) had a treatment duration of within 5 years. Out of the total participants, 183 (45.3%) had taken first generation antipsychotic; 103 (25.5%) of the participants were non-adherent and about 237 (58.7%) of the participants had mild medication side effects whereas nearly one third 133 (32.9%) had moderate side effects. About half of the participants 217 (53.7%) had experienced three or more stressful life events and 151 (37.4%) of the participants had poor social support (See Table 2 below).

**Table 2:** Description of clinical and psychosocial factors of participants with schizophrenia spectrum disorders in Comprehensive Specialized Hospitals, Amhara region, Ethiopia, 2020 (n = 404)

Variables	Frequency (404)	Percentage (%)
Clinical diagnosis		
Schizophrenia	346	85.6
Schizophreniform	9	2.2
Schizo-affective	26	6.4
Delusional	16	4
Brief psychotic	7	1.8
Age of onset		
< 20 years	44	10.9
≥ 20 years	360	89.1
Delayed treatment		
< 1 month	123	30.5
1–6 months	87	21.5
> 6 months	194	48.0
Duration on treatment		
Within 5 years	302	74.8
> 5 years	102	25.2
Types of medication taking		
First generation APS	183	45.3
Second generation APS	171	42.3
Combination of first and second generation APS	2	0.5
Others*	48	11.9
Previous suicidal ideation		
Yes	15	3.7
No	389	96.3
Comorbid of another mental illnesses		
Yes	92	22.8
No	312	77.2
Comorbid another medical illness		
Yes	47	11.6
No	357	88.4
Medication adherence		
Adherent	301	74.5
Non adherent	103	25.5
Medication side effect		
Mild side effect	237	58.7
Moderate side effect	133	32.9
Severe side effect	34	8.4
Social support		
Poor support	151	37.4
Moderate support	142	35.1
Severe support	111	27.5
Stressful event		
No stressful event	53	13.1
1or 2 stressful events	134	33.2
≥ 3 stressful events	217	53.7

* = Antipsychotic and antidepressants, antipsychotic and mood stabilizers; *APS* Antipsychotics

### Substance use

Out of the total participants, 78 (19.3%) used alcoholic products; of those 68 (16.8%) had alcohol abuse. Regarding khat abuse, 39 (9.6%) and 18 (4.5%) of the participants had moderate to substantial khat abuse respectively (See Table 3 below).

**Table 3:** Description of substance related factors of participants with schizophrenia spectrum disorders in Comprehensive Specialized Hospitals, Amhara region, Ethiopia, 2020 (n = 404)

Variables	Frequency (404)	Percentage (%)
Alcohol use		
Yes	78	19.3
No	326	80.7
Alcohol abuse		
Yes	68	16.8
No	10	2.5
Chat and cannabis abuse		
Absent abuse	342	84.7
Mild abuse	3	0.7
Moderate abuse	39	9.6
Substantial abuse	18	4.5
Severe abuse	2	0.5
Lifetime tobacco use		
Yes	35	8.7
No	369	91.3
Current tobacco use		
Yes	25	6.2
No	379	93.8

### Magnitude of lifetime relapses among peoples with schizophrenia spectrum disorders

**Fig. 2:**
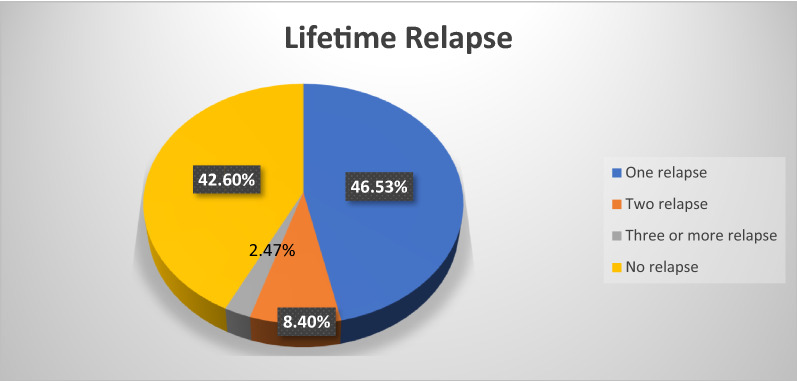
Rates of the magnitude of lifetime relapse among participants with schizophrenia spectrum disorders in the Comprehensive Specialized Hospitals, Amhara region, Ethiopia, 2020 (n = 404)

Among all participants, 232 (57.4%) had at least one-lifetime relapse with (95% CI = 53%– 62%); 188 (46.53%) had only one relapse and 34 (8.4%) had two relapses and 10 (2.47%) had more than three relapses (See Fig. 2 below). The lifetime relapse with the duration of a treatment since they began to take medication showed that 72 (31%) had experienced relapse within 24 months on treatment whereas, 112 (48.30%) and 48 (20.70%) had experienced the relapse within 25–60 months and greater than 60 months on the treatment respectively. Regarding treatment, 107 (46.12%) who had relapses are taking first-generation antipsychotics whereas 90 (38.8%) are taking second-generation antipsychotics. The remaining 33 (14.22%) of those who had relapses are taking antipsychotic plus mood stabilizer or antidepressant, and 2 (0.86%) of those with relapses are taking a combination of first and second-generation antipsychotics.

### Factors associated with lifetime relapse

The current study indicated that factors associated with lifetime relapse are short duration of treatment, medication non-adherence, having comorbid mental illnesses, and experiencing stressful life events.

The odds of developing relapse among participants who had a treatment duration of 1–5 years was 1.71 times (AOR = 1.71 95% CI = (1.05, 2.81) increased when compared to those who had a treatment duration of greater than five years. The odd of having relapse among participants who were non-adherent to their medication was 2.23 times (AOR = 2.23, 95% CI; (1.22, 4.07) higher as compared to those who were adherent. The odds of developing relapse in those who had comorbid mental illnesses and stressful life events were 1.84 times (AOR = 1.84, 95% CI; (1.06, 3.18), and 2.42 times (AOR = 2.42, 95%CI; (1.2, 4.66) increased than their counterparts respectively (See Table 4 below).

**Table 4:** Description of Bivariable and Multivariable binary logistic regression analysis showing association between lifetime relapse and associated factors among participants with schizophrenia spectrum disorders in Comprehensive Specialized Hospitals, Amhara, 2020 (404)

Explanatory variables	Relapse		COR	AOR	*P*-value
	Yes	No			
Sex					
Male	136	89	1.32 (0.88, 1.96)	1.23 (0.790, 1.92)	
Female	96	83			
Marital status					
Has spouse	45	23			
No spouse	187	149	0.64 (0.37, 1.108)	0.69 (0.38, 1.27)	
Income					
< 2044	110	93	0.76 (0.51, 1.13)	0.68 (0.43, 1.07)	
≥ 2044	122	79			
Duration on treatment					
Within 5 years	184	118	1.75 (1.11, 2.75)	1.71 (1.05, 2.81)	0.03
> 5 years	48	54			
Comorbid mental illness					
Yes	65	27	2.09 (1.26, 3.44)	1.84 (1.06, 3.18)	0.02
No	167	145			
Medication adherence					
Adherent	153	148			
Non adherent	79	24	3.18 (1.91, 5.30)	2.23 (1.22, 4.07)	0.009
Medication side effect					
Mild side effect	113	124	1		
Moderate side effect	94	39	2.64 (1.68, 4.15)	1.52 (0.9, 2.57)	
Severe side effect	25	9	3.04 (1.36, 6.80)	1.38 (0.52, 3.63)	
Stressful events					
Absent	24	29	1		
1 or 2	53	81	0.79 (0.41, 1.50)	0.84 (0.43, 1.66)	
≥ 3	155	62	3.02 (1.63, 5.59)	2.42 (1.2, 4.66)	0.008

Hosmer and Lemeshow test result was *P* value = 0.34

## Discussion

This study showed that 57.4% (95% CI = 53–62%) of people with schizophrenia spectrum disorders experienced relapse at least once in their life during their follow-up. Among those, 31% were had their lifetime relapse within 2 years of treatment whereas, 48.3% and 20.7% had their lifetime relapse within 2–5 years and after 5 years of treatment respectively. The lifetime magnitude of relapse in this study is in agreement with the study from Northern Ireland 56.3% [44] and South Africa 61.8% [2].

Many factors could attribute to the relapse and symptom exacerbation of schizophrenia spectrum disorders [14]. Non-adherence to antipsychotic medications is one of the important factors contributing to relapse [10, 22, 45–48]. This is consistent with our study results indicating that participants with medication non-adherence had more than twofold (AOR = 2.23) odds of experiencing relapse as compared to those who are adherent to their medication. This was supported by the study conducted in London [49], India [5], Pakistan [50], and Ethiopia [10, 19].

In another study from London, it was also reported that interruption of antipsychotic treatment was associated with a five-times increased risk of relapse [47]. Similarly, it was also evidenced by a systematic review as complete discontinuation of antipsychotic treatment is associated with a substantially increased risk of relapse [48]. This could be due to the reason that medication non-adherence is influenced by various factors such as; medication side-effect, lack of insight about their illness and treatment, and presence of comorbidity [51].

Non-adherence could result from psychotropic medication side effects [51]; a study from Butajira, Ethiopia also stated that side effects of medications as a reason for non-adherence [52]. Specific adverse effects have a higher impact on adherence than other factors [53]. The distress associated with persistent side effects to be an important contributor to non-adherence [54], non-adherence in return leads to relapse [19]. Additionally, lack of insight about their illness and medication might be a factor associated with psychotropic medication non-adherence [51, 55]. Increasing compliance with antipsychotic medication, educating patients and families about the illness and medication side effects should be the center of attention to prevent relapse and facilitate recovery.

Stressful life events and the presence of other primary comorbid mental illnesses are other factors that can lead to relapse [21, 50]. Our study indicated that participants who experienced three or more stressful life events had two times (AOR = 2.42) odds of experiencing relapse than their counterparts, and it was supported by a meta-analysis [56], and a study in Iraq [57].

Patients with relapses had more frequent relationship difficulties and personal stressors when compared with patients who had not relapsed [2]. This might be explained by the Stress-Vulnerability model which emphasizes that individuals with Schizophrenia have a biologically mediated vulnerability to stressful events that can result in acute psychosis; stress has a direct psychological effect on the body, cognitive and behavioral effects, and secondary effects by exacerbating illnesses, and delaying recovery [58]. Thus, in turn, cognitive vulnerability enhances susceptibility to stressful events could be considered as a reason for relapses. Therefore, cognitive disturbances should be assessed and coping skills training implemented because those patients have less effective coping skills [57].

The odds of developing relapse among participants with comorbidity of another primary mental illness was 1.84 times (AOR = 1.84) increased when compared with those who did not have a comorbidity of another primary mental illness and this study finding was supported by a study in Pakistan [50] and Ethiopia [21]. When there are co-morbidities of mental illnesses; psychotropic medication adherence is compromised. A report from two studies indicated that medication non-adherence was associated with patients having co-morbidities with their current psychiatric disorders [59, 60].

The presence of comorbid psychiatric conditions can themselves be risk factors for non-adherence. As an example, depression has been linked to non-adherence within patients with psychiatric diagnoses. Non-compliance because of comorbidity of psychiatric condition result through, the increased regimen complexity and increase concern about the potential adverse consequences of treatment [61], and non-compliance, in turn, leads to relapse [55, 62].

This indicates that medication adherence is important for the patient to control symptoms and prevent relapse. So, interventions aimed at improving medication adherence might help to mitigate relapse. Besides, screening for comorbidities of mental illnesses and stressful life events and providing specific treatment as well as assisting them to develop coping strategies would help prevent and/or decrease the risk of relapse.

The magnitude of lifetime relapse in this study is higher than the studies from Canada 35.7% [63], Hong Kong which is 48.1% [62], and previous studies done in Ethiopia; which was 43.3% in Addis Ababa [10] and 24.6% in Jimma [19]. The possible discrepancy for this might be due to the lower sample size in the study conducted in Canada and the use of a limited time frame to determine relapse in both Canada and Hong Kong studies. In a study conducted in Jimma, Ethiopia, the relapse was determined in the past six months. However, the magnitude of lifetime relapse was determined in the current study.

The other possible explanation for this variation might be the way relapse was determined; as in the current study, relapse was assessed by using both the clinical interview and the patient’s medical record review, whereas, only clinical interview and document review were used in a study done in Addis Ababa and Hong Kong respectively. Perhaps, it could be also the difference in the literacy level of the population and mental health services available in the developed countries.

Furthermore, the estimate of the magnitude of lifetime relapse of this study is lower than the reported study from Addis Ababa Amanuel mental health specialized hospital 87.69% [21]. This difference could be due to the majority of patients who visit Addis Ababa Hospitals were referred from different corners of the country for better treatment, and most likely they are chronic and had severe illness history.

Additionally, the possible reasons might be the variation in the study population; where, people with schizophrenia who are known to have a chronic course were included in the study conducted in Addis Ababa; in contrast, the current study, include all people with schizophrenia spectrum disorders which might contribute for the lower magnitude of relapse. Another reason might be lack of consensus in relapse definition could result in inconsistent findings of relapse in different studies [62].

In addition, the odds of developing relapse among patients who had a duration on treatment less than or equal to five years was 1.71 times (AOR = 1.71) higher than their counterparts. This finding was supported by a study in Finland [64] and a systematic review [65]. Indeed antipsychotics have beneficial effects in reducing relapses and long-lasting continuous antipsychotic treatment is good for the majority of patients with first-episode schizophrenia [64].

A systematic review indicated that an indefinite duration of maintenance treatment is required to prevent relapse, this is especially true in patients who had behaviors such as; suicide attempts, shown violent and aggressive behavior, and frequent relapses [65]. However, in doing so, the risk–benefit ratio of taking antipsychotic medications for a longer time should be considered and discussed with the patients and their attendants.

Even though it was not significantly associated with relapse, in this study, 94 (70.7%) of the participants with moderate and 25 (73.5%) of the participants with severe medication side effects had experienced lifetime relapse. A study done in Jimma, Ethiopia reported 29.3% of patients with psychotic relapse had experienced medication side effects and the medication side effects were associated with relapse [19].

### Strength and limitations of the study

The use of standardized and reliable tools and being the first multicenter study covered three large hospitals are the strengths of this study. However, the current study also had some limitations: first, due to the cross-sectional nature of the study the association between different factors and relapse may not show the causation; second, the information was collected through an interviewer-administered questioner, it could be prone to social desirability bias and recall bias. Third, poor documentation and difficulty in getting information for some subjective data may also underestimate the result. Finally, the relapse definition criteria for the time frame used solely for patients with schizophrenia was used for schizophrenia spectrum disorders.

## Conclusion

In the current study, the magnitude of relapse in schizophrenia spectrum disorders was as common as in previous reports. Nearly three in every five individuals with schizophrenia spectrum disorders had experienced symptom relapse during their lifetime after they began to take medication. Relapse was more common in individuals with other comorbid mental illnesses, treatment non-adherence, shorter duration of treatment, and stressful life events. Future studies should focus on the separate diagnosis of schizophrenia spectrum disorders (e.g. schizophrenia only) to estimate the relapse rate in specific diagnoses.

## Data Availability

The datasets used and/or analyzed during the current study are available from the corresponding authors on reasonable request.

## Abbreviations

AOR: Adjusted Odds Ratio
ASSIST: Alcohol, Smoking and Substance Involvement Screening Test
BSc: Bachelor of Science
CAGE: Cutdown, Annoyed, Guilty feeling, Eye opening
CI: Confidence Interval
DAST-10: Drug Abuse Screening Test-10
DCSH: Dessie Comprehensive Specialized Hospital
GASS: Glasgow Antipsychotic Side effect Scale
GCSH: Gondor Comprehensive Specialized Hospital
FHCSH: Felege Hiwot Comprehensive Specialized Hospital LTE: Life Threating Experiences
MARS: Medication Adherence Rating Scale
MSc: Master of Science
OPD: Outpatient Department
OSSS-3: Oslo Social Support Scale
SPSS: Statistical Package for Social Sciences
